# Genetic and Morphological Analyses Demonstrate That *Schizolecis guntheri* (Siluriformes: Loricariidae) Is Likely to Be a Species Complex

**DOI:** 10.3389/fgene.2018.00069

**Published:** 2018-03-02

**Authors:** Camila S. Souza, Guilherme J. Costa-Silva, Fábio F. Roxo, Fausto Foresti, Claudio Oliveira

**Affiliations:** ^1^Departamento de Morfologia, Universidade Estadual Paulista “Júlio de Mesquita Filho”, Instituto de Biociências de Botucatu, Botucatu, Brazil; ^2^Departamento de Biologia, Universidade Santo Amaro, São Paulo, Brazil

**Keywords:** coastal drainages, catfish, molecular identification, COI gene, GMYC model

## Abstract

*Schizolecis* is a monotypic genus of Siluriformes widely distributed throughout isolated coastal drainages of southeastern Brazil. Previous studies have shown that fish groups found in isolated river basins tend to differentiate over time in the absence of gene flow, resulting in allopatric speciation. In this study, we used partial sequences of the mitochondrial gene COI with the analysis of the General Mixed Yule Coalescent model (GMYC) and the Automatic Barcode Gap Discovery (ABGD) for single locus species delimitation, and a Principal Component Analysis (PCA) of external morphology to test the hypothesis that *Schizolecis guntheri* is a complex of species. We analyzed 94 samples of *S. guntheri* for GMYC and ABGD, and 82 samples for PCA from 22 coastal rivers draining to the Atlantic in southeastern Brazil from the Paraná State to the north of the Rio de Janeiro State. As a result, the GMYC model and the ABGD delimited five operational taxonomy units (OTUs – a nomenclature referred to in the present study of the possible new species delimited for the genetic analysis), a much higher number compared to the traditional alfa taxonomy that only recognizes *S. guntheri* across the isolated coastal rivers of Brazil. Furthermore, the PCA analysis suggests that *S. guntheri* is highly variable in aspects of external body proportions, including dorsal-fin spine length, pectoral-fin spine length, pelvic-fin spine length, lower caudal-fin spine length, caudal peduncle depth, anal width and mandibular ramus length. However, no exclusive character was found among the isolated populations that could be used to describe a new species of *Schizolecis*. Therefore, we can conclude, based on our results of PCA contrasting with the results of GMYC and ABGD, that *S. guntheri* represents a complex of species.

## Introduction

The distribution pattern of single fish species throughout independent hydrographic systems (i.e., current not connected rivers) is unusual among fishes of the Atlantic rainforest rivers (Menezes et al., 2007) as well as among members of Otothyrinae (Reis et al., 2003). Recently, several genetic studies focusing on freshwater fishes, such as *Rineloricaria* (Costa-Silva et al., 2015), *Curimatopsis* (Melo et al., 2016), *Piabina* (Pereira et al., 2011), and *Astyanax* (Ornelas-Garcia et al., 2008), have shown that species of these groups may present large discontinuities in their distribution patterns with high genetic divergences, but with low morphological variability among geographically isolated populations. These results suggest that these groups may represent a complex of species –, i.e., *they are constituted by two or more morphological variable species that are erroneously classified (and hidden) under one species name* (Brown et al., 1995). Usually, studies focused on morphology alone are inadequate to recognize species complex. Integrative studies using molecular markers (e.g., DNA sequencing and allozymes), in addition to morphological comparison, are more powerful for the recognition of possible new species in such complex groups (Sytsma and Schaal, 1985).

The fast development of DNA sequencing and advances in molecular techniques in the last few years have been effective in recognizing species in several organism groups – birds (e.g., Tavares et al., 2011; Saitoh et al., 2015), fishes (e.g., Ward et al., 2005; Pereira et al., 2011, 2013; Roxo et al., 2012, 2015; Shimabukuro-Dias et al., 2016), insects (e.g., Hebert et al., 2004; Versteirt et al., 2015; Batovska et al., 2016), mammals (e.g., Borisenko et al., 2008; Li et al., 2015), plants (Kress et al., 2005; Lahaye et al., 2008; Braukmann et al., 2017), fungi (Begerow et al., 2010; Kelly et al., 2011), archaea (Bates et al., 2011), and bacteria (Sogin et al., 2006). The use of DNA sequences combined with several analytical methods, such as General Mixed Yule Coalescent (GMYC; Pons et al., 2006) and Automatic Barcode Gap Discovery (ABGD; Puillandre et al., 2012), support species delineation with single-locus data. The GMYC method is based on a likelihood method that seeks to determine the threshold between speciation and coalescent events from an ultrametric gene tree, whereas ABGD methods use the gap among organisms belonging to the same species and organisms from different species as a limit to species delimitation.

*Schizolecis* was described by Britski and Garavello (1984), being the species-type *Microlepidogaster guntheri* Miranda Ribeiro (1918). Currently *Schizolecis guntheri* is the only species of *Schizolecis*. This species is a descendant of a very ancient lineage that arose during the Middle Eocene approximately 42 Mya (Roxo et al., 2014), and inhabits small to median size streams with rocky and sandy bottoms, mostly in shallows and backwaters up to 30 cm deep, with slow water flow (Burgess, 1989). The work conducted by Britski and Garavello (1984) detected morphological differences only related to orbits of the eyes, body depth and head depth among populations, but without enough evidence to support the hypothesis that some of the analyzed populations could represent a new species. Therefore, despite *Schizolecis guntheri* being widely distributed across adjacent and not connected Atlantic Coastal rivers from the north of Santa Catarina to the north of Rio de Janeiro States (Menezes et al., 2007), and present small morphological variations among isolated populations; the doubt of whether *S. guntheri* represents a complex of species still remains.

In the present study, we used genetic data of 94 samples of *Schizolecis guntheri* from 22 coastal rivers draining directly to the Atlantic in southeastern Brazil and employed analytical methods to support species delineation with single-locus data (GMYC and ABGD), and analyzed the body shape variation among isolated populations using a PCA to test whether this species represents a complex of species.

## Materials and Methods

### Morphological Analysis

Principal Component Analysis (PCA) (Jolliffe, 2002) was used to check the external morphology variation among 82 samples of *Schizolecis guntheri* among regions of genetic groups (Supplementary Table S3) using the program Past version v1.28 (Hammer et al., 2004). Landmarks distances followed those originally proposed by Carvalho and Reis (2009), and they were measured for adult specimens (>26.2 mm SL). Prior to the PCA analysis, we followed the method of Dryden and Mardia (1998) to minimize body size influence on morphometric data. We normalized the first two coordinate dimensions, divided all coordinate values by the centroid size for each specimen, and conducted a Procrustes superimposition of the left half to a mirrored version of the right half. After that we also log transformed the data for base 10. The PCA loadings are presented in **Table 1**.

**Table 1:** Variable loadings in the first and second axes of the size-free principal component analysis (Axis 1 and Axis 2) of samples of *Schizolecis guntheri*.

	Axis 1	Axis 2
Standard length	0.03469	0.01922
Predorsal length	0.05589	0.05622
Preanal length	–0.0378	–0.08091
Head length	0.1271	0.0697
Cleithral width	0.1482	0.1679
Dorsal-fin spine length	**0.3355**	–0.2164
Base of dorsal-fin length	0.1576	0.002704
Thorax length	0.1218	0.01957
Pectoral-fin spine length	**0.3305**	–0.1625
Abdomen length	0.1143	0.1715
Pelvic-fin spine length	**0.3478**	0.1035
Anal-fin spine length	0.2795	–0.2604
Lower caudal-fin spine length	**0.5282**	–0.3743
Caudal peduncle depth	0.1384	**0.3224**
Caudal peduncle length	–0.04707	0.05234
Anal width	**0.3044**	0.287
Snout-opercle length	0.07479	0.1523
Head width	0.1181	0.14
Head depth	0.1298	0.2021
Snout length	0.07969	0.153
Interorbital width	0.08071	0.1185
Orbital diameter	0.01212	0.2747
Suborbital depth	0.1547	0.1987
Mandibular ramus length	0.127	**0.4584**

*Bold values represent the character with the highest variations.*

### Taxon Sampling for Genetic Analysis

**FIGURE 1:**
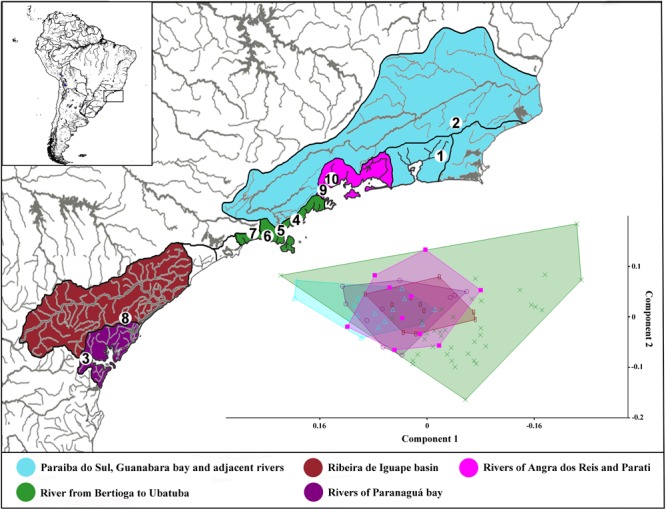
Image showing the map of coastal rivers of southeastern of Brazil following the paleodrainages inference of Thomaz et al. (2015), and adding the rivers of Guanabara Bay, the Paraiba do Sul basin and adjacent rivers. Numbers in the maps represents collection sites: **1** – Itaboraí-RJ; **2** – Bom Jardim-RJ; **3** – Morretes-PR; **4** – Ubatuba-SP; **5** – Caraguatatuba-SP; **6** – São Sebastião-SP; **7** – Bertioga-SP; **8** – Cajati-SP; **9** – Parati-RJ; and **10** – Angra dos Reis-RJ. Each number can represent more than one collection site.

We analyzed 94 *Schizolecis guntheri* specimens from 22 coastal rivers draining directly to the Atlantic, from the south of Paraná to the north of Rio de Janeiro States (**Figure 1**), almost comprising the entire distribution of this species. Information about the sample used in the present study is available in Bold Systems with the accession numbers for individuals present in Supplementary Table S1. The vouchers and tissues are deposited in the fish collection of the LBP – Laboratório de Biologia e Genética de Peixes, Universidade Estadual Paulista, Botucatu, São Paulo, Brazil. A sequence from one additional species of Loricariidae (*Hypostomus ancistroides*) was used as an outgroup to root our tree.

The abbreviation OTU (operational taxonomical unit) was used in the text to refer to the possible new species of the molecular analysis of the GMYC model and ABGD method (*sensu* Blaxter et al., 2005). This last author defined the term OTU to refer to genetic clusters of unknown organisms grouped by DNA sequences.

### Ethics Statement

All fishes collected for this study were collected in accordance with Brazilian laws, under a scientific collection license in the name of Dr. Claudio Oliveira (SISBIO). Furthermore, our laboratory has special federal permission to keep animals and tissues from a public collection under our care. To work with the animals, we followed all the ethical prescriptions stated by our internal committee of ethics (protocol number 388), called the “Comissão de Ética na Experimentação Animal” (CEEA), involving animal experiments that were *approved* for this study. After collection, animals were anesthetized with benzocaine, a piece of muscle tissue was extracted from the right side of the body and preserved in 95% ethanol. Specimens were fixed in 10% formalin for 2 weeks, and then transferred to 70% ethanol for permanent storage.

### DNA Extraction, Amplification, and Sequencing

We conducted the total genomic DNA extraction using the protocol described by Ivanova et al. (2006). Partial sequences of the cytochrome oxidase C subunit I (COI) gene were amplified (approximately 655 bp) using the primers Fish F1 and Fish R1 (Ward et al., 2005). Amplifications were performed in a total reaction mixture volume of 12.5 μl. Each reaction includes 1.25 μl of 10 X Buffer, 0.25 μl of MgCl_2_ (50 mM), 0.2 μl dNTPs (2 mM), 0.5 μl of each primer (5 mM), 0.1 μl of Pht Taq DNA polymerase (Phoneutria Biotecnologia e Serviços Ltda, Belo Horizonte, Brazil), 1 μl of genomic DNA (200 ng) and 8.7 ml ddH_2_O. The conditions for each PCR reaction consisted of an initial denaturation (5 min at 94°C), followed by 30 cycles of chain denaturation (40 s at 94°C), primer hybridization (30 s at 50–54°C), nucleotide extension (1 min at 68°C, considering the optimum temperature of the Pht Tap DNA polymerase) and final extension (8 min at 72°C, to stabilize the reaction). The amplified products were checked on 1% agarose gels and then purified using ExoSap-IT (USB Corporation, Cleveland, OH, United States) following the manufacturer’s instructions. We accomplished the sequencing reactions using the BigDye TM Terminator v 3.1 Cycle Sequencing Ready Reaction Kit (Applied Biosystems, Austin, TX, United States) and purified again by ethanol precipitation. DNA sequencing was conducted in an ABI 3130 DNA Analyzer automatic sequencer (Applied Biosystems, Foster City, CA, United States).

### Genetic Analysis

The consensus sequences were obtained using the program Geneious 7.1.4^1^ (Kearse et al., 2012) and the alignment was generated with the algorithm Muscle (Edgar, 2004) under default parameters. To evaluate the occurrence of substitution saturation in our molecular data, we estimated whether the Iss (index of substitution saturation) is significantly lower than Iss.cAsym (assuming asymmetrical topology) using the method described by Xia et al. (2003) with the software DAMBE 5.3.38 (Xia, 2013). After the identification of the OTUs by the GMYC model and ABGD analysis, we calculated the genetic variation within and among the OTUs delimited by each method using the Kimura-2-parameter (K2P) model in the MEGA v.6.06 software (Tamura et al., 2013).

### GMYC Model

The GMYC method requires an ultrametric tree as input for the analysis. To estimate the ultrametric tree, we used Beast v1.8.2 (Drummond et al., 2012), employing an uncorrelated lognormal relaxed clock and birth-death speciation process, and the General Time Reversible (GTR) model (Lanave et al., 1984; Tavaré, 1986). The Bayesian topology reconstruction started with a UPGMA tree and the Markov Chain Monte Carlo (MCMC) method was performed for 100 million generations; a tree was sampled for every 20,000 generations. We used the software Tracer v1.6 (Rambaut et al., 2014) to check the convergence of the values. All sampled topologies beneath the asymptote (20,000,000 generations) were discarded as part of a burn-in procedure, and the remaining trees were used to construct a 90% majority-rule consensus tree using Tree Annotator v1.8.2 (Drummond et al., 2012). The GMYC analysis was performed with the package Species Limits by Threshold Statistics (“splits”) (Fujisawa and Barraclough, 2013) using R v3.0.0 (R Development Core Team, 2014) that only includes the ingroup (the outgroup *Hypostomus ancistroides* was excluded).

### ABGD Analysis

Automatic Barcode Gap Discovery analysis (Puillandre et al., 2012), was processed using the “graphic” web version available at http://wwwabi.snv.jussieu.fr/public/abgd/abgdweb.html, under the default parameters of *P*_min_ = 0.001 to *P*_max_ = 0.1, steps = 10, X (relative gap width) = 1.5, Nb bins (for distance distribution) = 20, and the Kimura (K80) molecular model. For this analysis, the external group (*H. ancistroides*) was excluded from the input data.

## Results

### Morphological Analysis

The first (PC1) and second (PC2) principal component axis of our analysis explained 28.1% and 12.4%, respectively, of variation in body shape for all analyzed *Schizolecis guntheri* specimens. The variation is partly distributed within populations and partly between populations, and apparently, it represents a continuous distribution of external morphology, as we can observe in the PCA scatter plot (**Figure 1**). Our results also showed that the measures with greater variations were, respectively: dorsal-fin spine length, pectoral-fin spine length, pelvic-fin spine length, lower caudal-fin spine length, caudal peduncle depth, anal width and mandibular ramus length, as we can observe in the PCA loading values (**Table 1**).

### Statistics of the DNA Matrix

We obtained a matrix with 533 characters, 230 of which were variable. The nucleotide frequencies were A (25.4%), G (16.7%), T (29.3%), and C (28.7%). No insertions, deletions, stop codons or contamination in the sequences were detected. The data were not saturated considering that the Iss.cAsym values are higher than the Iss for the different numbers of NumOTU analyzed (4 OTUs: Iss = 0.187, Iss.cAsym = 0.763, *P* = 0; 8 OTUs: Iss = 0. 177, Iss.cAsym = 0.643, *P* = 0; 16 OTUs: Iss = 0.179, Iss.cAsym = 0.516, *P* = 0; and 32 OTUs: Iss = 0.180, Iss.cAsym = 0.383, *P* = 0) in the total matrix (all molecular characters including gaps).

### GMYC Model and ABGD Analysis

**FIGURE 2:**
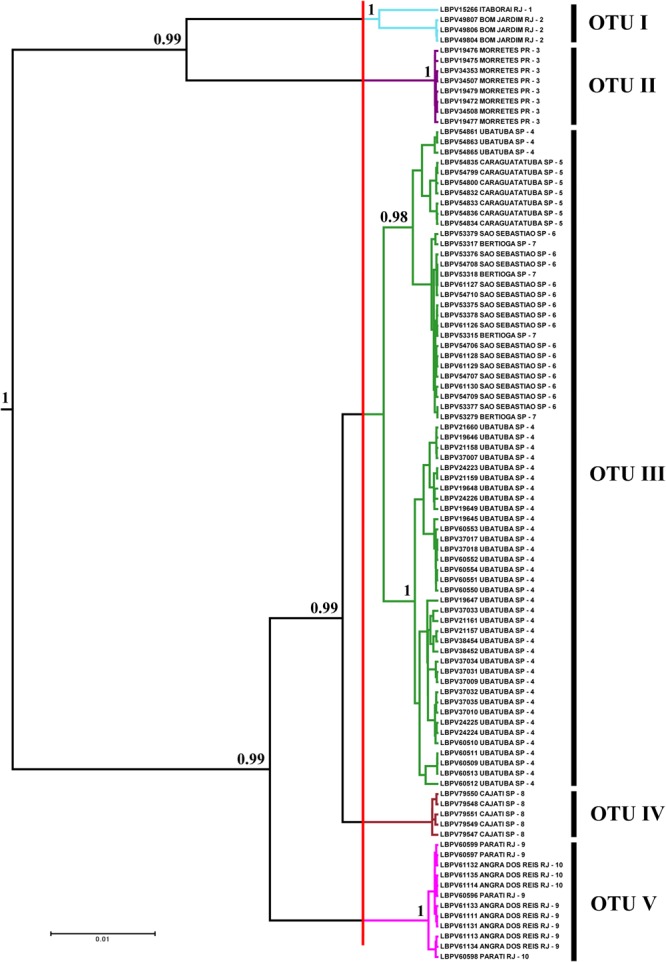
Bayesian tree showing the distribution of specimens by OTUs selected through GMYC analysis. Numbers after branches are posterior probabilities. Values below 0.95 are not shown. The vertical red line represents the threshold time (–0.0055687) – the limit between species and population according to the GMYC model in the tree. The colors of the branches to the right of the red line correspond to the colors of the paleodrainages in **Figure 1**, and numbers after are local to collection sites also in **Figure 1**. LBPV is the code for samples of the fish collection of the *Laboratório de Biologia e Genética de Peixes* on the Bold systems site.

The phylogenetic reconstruction resulted in a tree with high values of posterior probability among the clades (>95%), highlighting the existence of five lineages within *Schizolecis guntheri* (**Figure 2**). The analysis of species delimitation using the GMYC model under a phylogenetic tree estimated using a Birth–Death model prior of branching rates showed that the found threshold time was -0.0055687, indicating the time before which all nodes reflect diversification events and after which all nodes in the tree reflect coalescent events. The likelihood of the null model was 958.2078 and the maximum likelihood of the GMYC model was 962.0088. The standard log-likelihood ratio test is used to assess whether the alternative model (GMYC model) provides a better fit than the null model in the branching process (see Goldman, 1993 and Pons et al., 2006 for a better explanation about how the GMYC model assesses species delimitation).

The analysis of ABGD partitioned the data from 19 groups (*P*_min_ = 0.0010003) to 3 groups (*P*_max_ = 0.021544). The value of *P* = 0.004642 delimited five groups, the same number of groups delimited by the GMYC model (see Supplementary Table S1). Therefore, the OTUs for *Schizolecis guntheri* were divided following the genetic delimitation of five clusters (OTUs) according to the results of both genetic methods, and named according to their localities: OTU I – Itaboraí-RJ and Bom Jardim-RJ; OTU II – Morretes-PR; OTU III – Ubatuba-SP, Caraguatatuba-SP, São Sebastião-SP and Bertioga-SP; OTU IV – Cajati-SP; and OTU V – Angra dos Reis and Parati (**Figures 1, 2** and Supplementary Table S1). The values of genetic distance among the five OTUs ranged from 1.1% (OTU III and OTU IV) to 8.7% (OTU II and OTU V) (**Table 2**).

**Table 2:** Genetic divergences based on the Kimura-2-parameter (K2P) nucleotide model for OTUs delimitated by GMYC analysis.

	OTU I	OTU II	OTU III	OTU IV	OTU V
OTU I	**1.1 ± 0.3**				
OTU II	6.4 ± 1.2	**0**			
OTU III	8.1 ± 1.3	8.6 ± 1.4	**0.8 ± 0.3**		
OTU IV	8.0 ± 1.4	8.4 ± 1.5	1.1 ± 0.4	**0**	
OTU V	8.0 ± 1.4	8.7 ± 1.5	2.3 ± 0.7	1.9 ± 0.6	**0**

*In the main diagonal are the values of intragroup genetic divergences highlighted in bold. Below the main diagonal are the values of intergroup (OTUs) genetic divergences. The values are shown in percentages.*

## Discussion

The results of the Bayesian and GMYC analyses (**Figure 2**) of the samples from 22 coastal rivers localities draining directly to the Atlantic of southeastern Brazil (**Figure 1**) highlighted the existence of five monophyletic and highly statistically supported (>95%) lineages (OTUs *sensu* Blaxter et al., 2005) within *Schizolecis guntheri* (**Figure 2**), the same result found by the ABGD method (Supplementary Table S2) that also divided the data into five clusters (or five OTUs). Several authors (e.g., Hänflig and Brandl, 1998; Paggi et al., 1998; Baric and Sturmbauer, 1999; Ferguson, 2002) have summarized the arguments for using genetic divergence for identifying separate species. According to these authors, sufficient genetic distance indicates reproductive isolation between probable/possible species (*sensu* Mayr, 1963) that gradually accumulates genetic differences between lineages, and after long-time periods, accumulates sufficient genetic divergence so that a speciation event could be detected.

However, despite the similarity of the results of the GMYC and ABGD analysis methods in the present study, if we apply the 2% threshold of interspecific genetic divergence as a limit among population and species (as proposed by Smith et al., 2005) the OTU III and OTU IV with 1.1% and OTU IV and OTU V with 1.9% (**Table 2**) should be considered members of the same cluster. The different number of species delimited for different molecular methods is also a problematic question in species delimitation using single locus genes (Pons et al., 2006). However, the two analytical molecular methods used in the present study (e.g., GMYC and ABGD) resulted in the same numbers of OTUs (i.e., five OTUs) and the application of these methods has been encouraging, highlighting a hidden genetic diversity in several neotropical fish species (e.g., Costa-Silva et al., 2015; Roxo et al., 2015; Melo et al., 2016). Furthermore, molecular studies in several Neotropical fish groups (e.g., Pereira et al., 2011; Costa-Silva et al., 2015; Melo et al., 2016) have shown that widely distributed nominal species isolated in independent hydrographic systems can sometimes represent species complex (i.e., species with continuous morphological variation, but discontinuous variation in genetic analysis) and the combined usage of DNA barcoding and morphological data has provided support to recognize and describe possible new species (Melo et al., 2011; Amaral et al., 2013; Silva et al., 2013).

**FIGURE 3:**
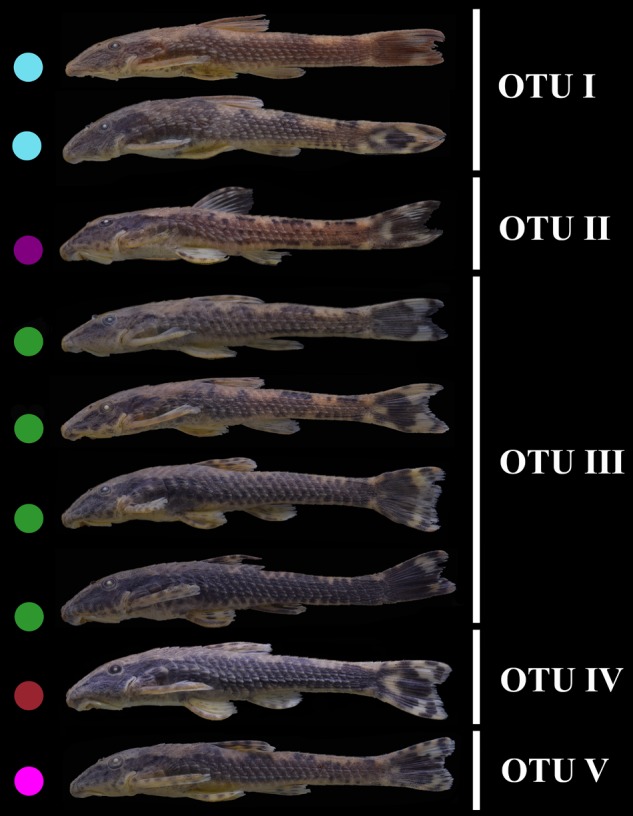
Pictures showing the variation in external morphology among *Schizolecis guntheri* populations. Colored circles represent the paleodrainages proposed by Thomaz et al. (2015) shown in **Figure 1**.

The results of *Schizolecis guntheri* morphological analysis exhibits high external morphological variation across its distribution, including color pattern (**Figures 1, 3**), but especially in morphometric characteristics, such as dorsal-fin spine length, pectoral-fin spine length, pelvic-fin spine length, lower caudal-fin spine length, caudal peduncle depth, anal width and mandibular ramus length, as we can observe in the PCA loading values (**Table 1**). However, this diversity is partly distributed within populations and among populations, and represents a continuous variation in external morphology, as we can observe in the PCA scatter plot (**Figure 1**). This continuous morphological variation is typically found in a species complex (Brown et al., 1995). Therefore, we do not find any exclusive character to support a possible new species for any of the OTUs distributed across the isolated coastal drainages of the present study. A similar result was previously found by Britski and Garavello (1984). These authors found differences in the morphometric characters of eyes orbits, body depth and head depth, but no exclusive character that could support a new species of the genus *Schizolecis* among the analyzed samples.

De Queiroz (2007) argued that the confusion among species delimitation is associated with the fact that different methods, including different properties (molecular or morphological), are focused on different stages of the speciation process. Our results suggested different numbers of OTUs of *S. guntheri* delimited based on the GMYC model and ABGD (Genotypic Cluster) that recognized five monophyletic groups and the morphological analysis (Phenetic Cluster) that only recognized *S. guntheri* with a continuous variation in the external morphology, as shown by the PCA (typical results of a species complex). With the passing of time, two independent lineages develop and increasingly acquire different properties relative to each other –, i.e., they become phenetically distinct, reach the reciprocal monophyly, became ecologically distinct or reproductively incompatible. Before the recovery of the first estate, everybody will recognize that there is only one species, and after the acquisition of several estates, everyone will recognize two species. Otherwise, in between, there will be confusion. De Queiroz (2007) called the area where two groups of species come into conflict and the boundaries among species are unclear as the “gray zone.” On either side of the gray zone, there will be consistent agreement about the species number, but the “gray zone” has conflict. Therefore, the conflict among different numbers of recognized species between genetic methods (GMYC and ABGD) and the morphological analysis in *S. guntheri* could be associated with the fact that this species is in the gray zone suggested by De Queiroz (2007). Furthermore, a species complex could be interpreted as species that are in the gray zone (e.g., molecularly distinguished but morphologically undistinguished).

Therefore, considering the lack of phenotypic discontinuities and the presence of relatively high levels of genetic divergence among some local populations of *Schizolecis guntheri*, we hypothesize that this species may represent a species complex, like suggested for other freshwater fish species (Pereira et al., 2011; Bellafronte et al., 2013; Marques et al., 2013).

## Author Contributions

CS, CO, GC-S, and FR designed the ideas of the research. CS collected data. CS, GC-S, and FR performed the analyses. CS, CO, GC-S, and FR contributed to the writing of the manuscript. FF provided physical structure of the laboratory to develop the work.

## Conflict of Interest Statement

The authors declare that the research was conducted in the absence of any commercial or financial relationships that could be construed as a potential conflict of interest.

## Footnotes

1 http://www.geneious.com
