# Supplementary material for: Genetic and Morphological Analyses Demonstrate That Schizolecis guntheri (Siluriformes: Loricariidae) Is Likely to Be a Species Complex
Source: Front Genet. 2018 Mar 2;9:69. doi: 10.3389/fgene.2018.00069 (PMC5841391; doi:10.3389/fgene.2018.00069)
Supplement: Supplementary file 1 [file Table_1.DOCX]

**Supplementary table 1.** Sequences of *Schizolecis guntheri* available in Boldsystems.

| Fish No | Process ID Bold Systems | Collection No | Species | Municipality/State | Geographic coordinates | |
| --- | --- | --- | --- | --- | --- | --- |
| LBPV15266 | LBCR057-16 | LBP2513 | *Schizolecis guntheri* | Itaboraí/RJ | -22.525 | -42.688 |
| LBPV49804 | LBCR092-16 | LBP10759 | *Schizolecis guntheri* | Bom Jardim/RJ | -22.227 | -42.444 |
| LBPV49806 | LBCR093-16 | LBP10759 | *Schizolecis guntheri* | Bom Jardim/RJ | -22.227 | -42.444 |
| LBPV49807 | LBCR094-16 | LBP10759 | *Schizolecis guntheri* | Bom Jardim/RJ | -22.227 | -42.444 |
| LBPV61111 | LBCR082-16 | LBP14427 | *Schizolecis guntheri* | Angra dos Reis/RJ | -22.962 | -44.559 |
| LBPV61113 | LBCR027-16 | LBP14427 | *Schizolecis guntheri* | Angra dos Reis/RJ | -22.962 | -44.559 |
| LBPV61114 | LBCR026-16 | LBP14427 | *Schizolecis guntheri* | Angra dos Reis/RJ | -22.962 | -44.559 |
| LBPV61131 | LBCR083-16 | LBP14700 | *Schizolecis guntheri* | Angra dos Reis/RJ | -22.962 | -44.559 |
| LBPV61132 | LBCR029-16 | LBP14700 | *Schizolecis guntheri* | Angra dos Reis/RJ | -22.962 | -44.559 |
| LBPV61133 | LBCR031-16 | LBP14700 | *Schizolecis guntheri* | Angra dos Reis/RJ | -22.962 | -44.559 |
| LBPV61134 | LBCR030-16 | LBP14700 | *Schizolecis guntheri* | Angra dos Reis/RJ | -22.962 | -44.559 |
| LBPV61135 | LBCR028-16 | LBP14700 | *Schizolecis guntheri* | Angra dos Reis/RJ | -22.962 | -44.559 |
| LBPV60596 | LBCR035-16 | LBP14421 | *Schizolecis guntheri* | Parati-RJ | -23.123 | -44.729 |
| LBPV60597 | LBCR034-16 | LBP14421 | *Schizolecis guntheri* | Parati-RJ | -23.123 | -44.729 |
| LBPV60598 | LBCR033-16 | LBP14421 | *Schizolecis guntheri* | Parati-RJ | -23.123 | -44.729 |
| LBPV60599 | LBCR032-16 | LBP14421 | *Schizolecis guntheri* | Parati-RJ | -23.123 | -44.729 |
| LBPV19645 | LBCR040-16 | LBP2988 | *Schizolecis guntheri* | Ubatuba-SP | -23.408 | -45.073 |
| LBPV19646 | LBCR041-16 | LBP2988 | *Schizolecis guntheri* | Ubatuba-SP | -23.408 | -45.073 |
| LBPV19647 | LBCR038-16 | LBP2988 | *Schizolecis guntheri* | Ubatuba-SP | -23.408 | -45.073 |
| LBPV19648 | LBCR042-16 | LBP2988 | *Schizolecis guntheri* | Ubatuba-SP | -23.408 | -45.073 |
| LBPV19649 | LBCR088-16 | LBP2988 | *Schizolecis guntheri* | Ubatuba-SP | -23.408 | -45.073 |
| LBPV21157 | LBCR073-16 | LBP3546 | *Schizolecis guntheri* | Ubatuba-SP | -23.405 | -45.064 |
| LBPV21158 | LBCR086-16 | LBP3546 | *Schizolecis guntheri* | Ubatuba-SP | -23.405 | -45.064 |
| LBPV21159 | LBCR087-16 | LBP3546 | *Schizolecis guntheri* | Ubatuba-SP | -23.405 | -45.064 |
| LBPV21161 | LBCR012-16 | LBP3546 | *Schizolecis guntheri* | Ubatuba-SP | -23.405 | -45.064 |
| LBPV21160 | LBCR085-16 | LBP3546 | *Schizolecis guntheri* | Ubatuba-SP | -23.405 | -45.064 |
| LBPV24223 | LBCR043-16 | LBP4402 | *Schizolecis guntheri* | Ubatuba-SP | -23.4014 | -45.065 |
| LBPV24224 | LBCR036-16 | LBP4402 | *Schizolecis guntheri* | Ubatuba-SP | -23.4014 | -45.065 |
| LBPV24225 | LBCR037-16 | LBP4402 | *Schizolecis guntheri* | Ubatuba-SP | -23.4014 | -45.065 |
| LBPV24226 | LBCR044-16 | LBP4402 | *Schizolecis guntheri* | Ubatuba-SP | -23.4014 | -45.065 |
| LBPV37007 | LBCR039-16 | LBP7901 | *Schizolecis guntheri* | Ubatuba-SP | -23.4 | -45.069 |
| LBPV37009 | LBCR071-16 | LBP7901 | *Schizolecis guntheri* | Ubatuba-SP | -23.4 | -45.069 |
| LBPV37010 | LBCR084-16 | LBP7901 | *Schizolecis guntheri* | Ubatuba-SP | -23.4 | -45.069 |
| LBPV37017 | LBCR070-16 | LBP7911 | *Schizolecis guntheri* | Ubatuba-SP | -23.395 | -45.121 |
| LBPV37018 | LBCR002-16 | LBP7911 | *Schizolecis guntheri* | Ubatuba-SP | -23.395 | -45.121 |
| LBPV37031 | LBCR006-16 | LBP7921 | *Schizolecis guntheri* | Ubatuba-SP | -23.446 | -45.09 |
| LBPV37032 | LBCR007-16 | LBP7921 | *Schizolecis guntheri* | Ubatuba-SP | -23.446 | -45.09 |
| LBPV37033 | LBCR005-16 | LBP7921 | *Schizolecis guntheri* | Ubatuba-SP | -23.446 | -45.09 |
| LBPV37034 | LBCR004-16 | LBP7921 | *Schizolecis guntheri* | Ubatuba-SP | -23.446 | -45.09 |
| LBPV37035 | LBCR003-16 | LBP7921 | *Schizolecis guntheri* | Ubatuba-SP | -23.446 | -45.09 |
| LBPV38452 | LBCR075-16 | LBP8244 | *Schizolecis guntheri* | Ubatuba-SP | -23.404 | -45.064 |
| LBPV38454 | LBCR074-16 | LBP8244 | *Schizolecis guntheri* | Ubatuba-SP | -23.404 | -45.064 |
| LBPV60509 | LBCR009-16 | LBP14397 | *Schizolecis guntheri* | Ubatuba-SP | -23.393 | -45.028 |
| LBPV60510 | LBCR010-16 | LBP14397 | *Schizolecis guntheri* | Ubatuba-SP | -23.393 | -45.028 |
| LBPV60511 | LBCR011-16 | LBP14397 | *Schizolecis guntheri* | Ubatuba-SP | -23.393 | -45.028 |
| LBPV60512 | LBCR072-16 | LBP14397 | *Schizolecis guntheri* | Ubatuba-SP | -23.393 | -45.028 |
| LBPV60513 | LBCR008-16 | LBP14397 | *Schizolecis guntheri* | Ubatuba-SP | -23.393 | -45.028 |
| LBPV60550 | LBCR089-16 | LBP14410 | *Schizolecis guntheri* | Ubatuba-SP | -23.355 | -44.951 |
| LBPV60551 | LBCR090-16 | LBP14410 | *Schizolecis guntheri* | Ubatuba-SP | -23.355 | -44.951 |
| LBPV60552 | LBCR046-16 | LBP14410 | *Schizolecis guntheri* | Ubatuba-SP | -23.355 | -44.951 |
| LBPV60553 | LBCR047-16 | LBP14410 | *Schizolecis guntheri* | Ubatuba-SP | -23.355 | -44.951 |
| LBPV60554 | LBCR045-16 | LBP14410 | *Schizolecis guntheri* | Ubatuba-SP | -23.355 | -44.951 |
| LBPV54861 | LBCR056-16 | LBP14391 | *Schizolecis guntheri* | Ubatuba-SP | -23.484 | -45.173 |
| LBPV54863 | LBCR091-16 | LBP14391 | *Schizolecis guntheri* | Ubatuba-SP | -23.484 | -45.173 |
| LBPV54865 | LBCR055-16 | LBP14391 | *Schizolecis guntheri* | Ubatuba-SP | -23.484 | -45.173 |
| LBPV54832 | LBCR050-16 | LBP14384 | *Schizolecis guntheri* | Caraguatatuba-SP | -23.564 | -45.309 |
| LBPV54833 | LBCR051-16 | LBP14384 | *Schizolecis guntheri* | Caraguatatuba-SP | -23.564 | -45.309 |
| LBPV54834 | LBCR052-16 | LBP14384 | *Schizolecis guntheri* | Caraguatatuba-SP | -23.564 | -45.309 |
| LBPV54835 | LBCR053-16 | LBP14384 | *Schizolecis guntheri* | Caraguatatuba-SP | -23.564 | -45.309 |
| LBPV54836 | LBCR054-16 | LBP14384 | *Schizolecis guntheri* | Caraguatatuba-SP | -23.564 | -45.309 |
| LBPV54799 | LBCR013-16 | LBP14372 | *Schizolecis guntheri* | Caraguatatuba-SP | -23.609 | -45.601 |
| LBPV54800 | LBCR049-16 | LBP14372 | *Schizolecis guntheri* | Caraguatatuba-SP | -23.609 | -45.401 |
| LBPV54706 | LBCR021-16 | LBP14342 | *Schizolecis guntheri* | São Sebastião-SP | -23.778 | -45.611 |
| LBPV54707 | LBCR018-16 | LBP14342 | *Schizolecis guntheri* | São Sebastião-SP | -23.778 | -45.611 |
| LBPV54708 | LBCR020-16 | LBP14342 | *Schizolecis guntheri* | São Sebastião-SP | -23.778 | -45.611 |
| LBPV54709 | LBCR015-16 | LBP14342 | *Schizolecis guntheri* | São Sebastião-SP | -23.778 | -45.611 |
| LBPV54710 | LBCR013-16 | LBP14342 | *Schizolecis guntheri* | São Sebastião-SP | -23.778 | -45.611 |
| LBPV53375 | LBCR014-16 | LBP14335 | *Schizolecis guntheri* | São Sebastião-SP | -23.765 | -45.684 |
| LBPV53376 | LBCR016-16 | LBP14335 | *Schizolecis guntheri* | São Sebastião-SP | -23.765 | -45.684 |
| LBPV53377 | LBCR017-16 | LBP14335 | *Schizolecis guntheri* | São Sebastião-SP | -23.765 | -45.684 |
| LBPV53378 | LBCR080-16 | LBP14335 | *Schizolecis guntheri* | São Sebastião-SP | -23.765 | -45.684 |
| LBPV53379 | LBCR019-16 | LBP14335 | *Schizolecis guntheri* | São Sebastião-SP | -23.765 | -45.684 |
| LBPV61126 | LBCR078-16 | LBP14433 | *Schizolecis guntheri* | São Sebastião-SP | -23.73 | -45.733 |
| LBPV61127 | LBCR081-16 | LBP14433 | *Schizolecis guntheri* | São Sebastião-SP | -23.73 | -45.733 |
| LBPV61128 | LBCR079-16 | LBP14433 | *Schizolecis guntheri* | São Sebastião-SP | -23.73 | -45.733 |
| LBPV61129 | LBCR077-16 | LBP14433 | *Schizolecis guntheri* | São Sebastião-SP | -23.73 | -45.733 |
| LBPV61130 | LBCR076-16 | LBP14433 | *Schizolecis guntheri* | São Sebastião-SP | -23.73 | -45.733 |
| LBPV53279 | LBCR024-16 | LBP14310 | *Schizolecis guntheri* | Bertioga-SP | -23.774 | -45.956 |
| LBPV53315 | LBCR025-16 | LBP14319 | *Schizolecis guntheri* | Bertioga-SP | -23.723 | -45.875 |
| LBPV53317 | LBCR022-16 | LBP14319 | *Schizolecis guntheri* | Bertioga-SP | -23.723 | -45.875 |
| LBPV53318 | LBCR023-16 | LBP14319 | *Schizolecis guntheri* | Bertioga-SP | -23.723 | -45.875 |
| LBPV79547 | LBCR065-16 | LBP20209 | *Schizolecis guntheri* | Cajati-SP | -24.802 | -48.238 |
| LBPV79548 | LBCR066-16 | LBP20209 | *Schizolecis guntheri* | Cajati-SP | -24.802 | -48.238 |
| LBPV79549 | LBCR067-16 | LBP20209 | *Schizolecis guntheri* | Cajati-SP | -24.802 | -48.238 |
| LBPV79550 | LBCR068-16 | LBP20209 | *Schizolecis guntheri* | Cajati-SP | -24.802 | -48.238 |
| LBPV79551 | LBCR069-16 | LBP20209 | *Schizolecis guntheri* | Cajati-SP | -24.802 | -48.238 |
| LBPV19472 | LBCR058-16 | LBP3241 | *Schizolecis guntheri* | Morretes-PR | -25.508 | -48.876 |
| LBPV19475 | LBCR061-16 | LBP3241 | *Schizolecis guntheri* | Morretes-PR | -25.508 | -48.876 |
| LBPV19476 | LBCR095-16 | LBP3241 | *Schizolecis guntheri* | Morretes-PR | -25.508 | -48.876 |
| LBPV19477 | LBCR059-16 | LBP3241 | *Schizolecis guntheri* | Morretes-PR | -25.508 | -48.876 |
| LBPV19479 | LBCR060-16 | LBP3241 | *Schizolecis guntheri* | Morretes-PR | -25.508 | -48.876 |
| LBPV34353 | LBCR064-16 | LBP7169 | *Schizolecis guntheri* | Morretes-PR | -25.441 | -48.874 |
| LBPV34507 | LBCR062-16 | LBP7169 | *Schizolecis guntheri* | Morretes-PR | -25.441 | -48.874 |
| LBPV34508 | LBCR063-16 | LBP7169 | *Schizolecis guntheri* | Morretes-PR | -25.441 | -48.874 |
| LBPV10887 | KM104509 | LBP2544 | *Hypostomus ancistroides* | Corumbataí-SP | - | - |
