# Supplementary material for: Genetic and Morphological Analyses Demonstrate That Schizolecis guntheri (Siluriformes: Loricariidae) Is Likely to Be a Species Complex
Source: Front Genet. 2018 Mar 2;9:69. doi: 10.3389/fgene.2018.00069 (PMC5841391; doi:10.3389/fgene.2018.00069)
Supplement: Supplementary file 2 [file Table_2.DOCX]

**Supplementary table 2.** -OTUs of *Schizolecis guntheri* populations resulted from the GMYC model and ABGD analysis.

| OTUs | Fish No | Process ID Bold Systems | Collection No | Species | Municipality/State |
| --- | --- | --- | --- | --- | --- |
| **OTU 1** | LBPV15266 | LBCR057-16 | LBP2513 | *Schizolecis guntheri* | Itaboraí-RJ |
| **OTU 1** | LBPV49804 | LBCR092-16 | LBP10759 | *Schizolecis guntheri* | Bom Jardim-RJ |
| **OTU 1** | LBPV49806 | LBCR093-16 | LBP10759 | *Schizolecis guntheri* | Bom Jardim-RJ |
| **OTU 1** | LBPV49807 | LBCR094-16 | LBP10759 | *Schizolecis guntheri* | Bom Jardim-RJ |
| **OTU 2** | LBPV19472 | LBCR058-16 | LBP3241 | *Schizolecis guntheri* | Morretes-PR |
| **OTU 2** | LBPV19475 | LBCR061-16 | LBP3241 | *Schizolecis guntheri* | Morretes-PR |
| **OTU 2** | LBPV19476 | LBCR095-16 | LBP3241 | *Schizolecis guntheri* | Morretes-PR |
| **OTU 2** | LBPV19477 | LBCR059-16 | LBP3241 | *Schizolecis guntheri* | Morretes-PR |
| **OTU 2** | LBPV19479 | LBCR060-16 | LBP3241 | *Schizolecis guntheri* | Morretes-PR |
| **OTU 2** | LBPV34353 | LBCR064-16 | LBP7169 | *Schizolecis guntheri* | Morretes-PR |
| **OTU 2** | LBPV34507 | LBCR062-16 | LBP7169 | *Schizolecis guntheri* | Morretes-PR |
| **OTU 2** | LBPV34508 | LBCR063-16 | LBP7169 | *Schizolecis guntheri* | Morretes-PR |
| **OTU 3** | LBPV19645 | LBCR040-16 | LBP2988 | *Schizolecis guntheri* | Ubatuba-SP |
| **OTU 3** | LBPV19646 | LBCR041-16 | LBP2988 | *Schizolecis guntheri* | Ubatuba-SP |
| **OTU 3** | LBPV19647 | LBCR038-16 | LBP2988 | *Schizolecis guntheri* | Ubatuba-SP |
| **OTU 3** | LBPV19648 | LBCR042-16 | LBP2988 | *Schizolecis guntheri* | Ubatuba-SP |
| **OTU 3** | LBPV19649 | LBCR088-16 | LBP2988 | *Schizolecis guntheri* | Ubatuba-SP |
| **OTU 3** | LBPV21157 | LBCR073-16 | LBP3546 | *Schizolecis guntheri* | Ubatuba-SP |
| **OTU 3** | LBPV21158 | LBCR086-16 | LBP3546 | *Schizolecis guntheri* | Ubatuba-SP |
| **OTU 3** | LBPV21159 | LBCR087-16 | LBP3546 | *Schizolecis guntheri* | Ubatuba-SP |
| **OTU 3** | LBPV21161 | LBCR012-16 | LBP3546 | *Schizolecis guntheri* | Ubatuba-SP |
| **OTU 3** | LBPV21660 | LBCR085-16 | LBP3546 | *Schizolecis guntheri* | Ubatuba-SP |
| **OTU 3** | LBPV24223 | LBCR043-16 | LBP4402 | *Schizolecis guntheri* | Ubatuba-SP |
| **OTU 3** | LBPV24224 | LBCR036-16 | LBP4402 | *Schizolecis guntheri* | Ubatuba-SP |
| **OTU 3** | LBPV24225 | LBCR037-16 | LBP4402 | *Schizolecis guntheri* | Ubatuba-SP |
| **OTU 3** | LBPV24226 | LBCR044-16 | LBP4402 | *Schizolecis guntheri* | Ubatuba-SP |
| **OTU 3** | LBPV37007 | LBCR039-16 | LBP7901 | *Schizolecis guntheri* | Ubatuba-SP |
| **OTU 3** | LBPV37009 | LBCR071-16 | LBP7901 | *Schizolecis guntheri* | Ubatuba-SP |
| **OTU 3** | LBPV37010 | LBCR084-16 | LBP7901 | *Schizolecis guntheri* | Ubatuba-SP |
| **OTU 3** | LBPV37017 | LBCR070-16 | LBP7911 | *Schizolecis guntheri* | Ubatuba-SP |
| **OTU 3** | LBPV37018 | LBCR002-16 | LBP7911 | *Schizolecis guntheri* | Ubatuba-SP |
| **OTU 3** | LBPV37031 | LBCR006-16 | LBP7921 | *Schizolecis guntheri* | Ubatuba-SP |
| **OTU 3** | LBPV37032 | LBCR007-16 | LBP7921 | *Schizolecis guntheri* | Ubatuba-SP |
| **OTU 3** | LBPV37033 | LBCR005-16 | LBP7921 | *Schizolecis guntheri* | Ubatuba-SP |
| **OTU 3** | LBPV37034 | LBCR004-16 | LBP7921 | *Schizolecis guntheri* | Ubatuba-SP |
| **OTU 3** | LBPV37035 | LBCR003-16 | LBP7921 | *Schizolecis guntheri* | Ubatuba-SP |
| **OTU 3** | LBPV38452 | LBCR075-16 | LBP8244 | *Schizolecis guntheri* | Ubatuba-SP |
| **OTU 3** | LBPV38454 | LBCR074-16 | LBP8244 | *Schizolecis guntheri* | Ubatuba-SP |
| **OTU 3** | LBPV60509 | LBCR009-16 | LBP14397 | *Schizolecis guntheri* | Ubatuba-SP |
| **OTU 3** | LBPV60510 | LBCR010-16 | LBP14397 | *Schizolecis guntheri* | Ubatuba-SP |
| **OTU 3** | LBPV60511 | LBCR011-16 | LBP14397 | *Schizolecis guntheri* | Ubatuba-SP |
| **OTU 3** | LBPV60512 | LBCR072-16 | LBP14397 | *Schizolecis guntheri* | Ubatuba-SP |
| **OTU 3** | LBPV60513 | LBCR008-16 | LBP14397 | *Schizolecis guntheri* | Ubatuba-SP |
| **OTU 3** | LBPV60550 | LBCR089-16 | LBP14410 | *Schizolecis guntheri* | Ubatuba-SP |
| **OTU 3** | LBPV60551 | LBCR090-16 | LBP14410 | *Schizolecis guntheri* | Ubatuba-SP |
| **OTU 3** | LBPV60552 | LBCR046-16 | LBP14410 | *Schizolecis guntheri* | Ubatuba-SP |
| **OTU 3** | LBPV60553 | LBCR047-16 | LBP14410 | *Schizolecis guntheri* | Ubatuba-SP |
| **OTU 3** | LBPV60554 | LBCR045-16 | LBP14410 | *Schizolecis guntheri* | Ubatuba-SP |
| **OTU 3** | LBPV54865 | LBCR055-16 | LBP14391 | *Schizolecis guntheri* | Ubatuba-SP |
| **OTU 3** | LBPV54863 | LBCR091-16 | LBP14391 | *Schizolecis guntheri* | Ubatuba-SP |
| **OTU 3** | LBPV54861 | LBCR056-16 | LBP14391 | *Schizolecis guntheri* | Ubatuba-SP |
| **OTU 3** | LBPV54832 | LBCR050-16 | LBP14384 | *Schizolecis guntheri* | Caraguatatuba-SP |
| **OTU 3** | LBPV54833 | LBCR051-16 | LBP14384 | *Schizolecis guntheri* | Caraguatatuba-SP |
| **OTU 3** | LBPV54834 | LBCR052-16 | LBP14384 | *Schizolecis guntheri* | Caraguatatuba-SP |
| **OTU 3** | LBPV54835 | LBCR053-16 | LBP14384 | *Schizolecis guntheri* | Caraguatatuba-SP |
| **OTU 3** | LBPV54836 | LBCR054-16 | LBP14384 | *Schizolecis guntheri* | Caraguatatuba-SP |
| **OTU 3** | LBPV54799 | LBCR013-16 | LBP14372 | *Schizolecis guntheri* | Caraguatatuba-SP |
| **OTU 3** | LBPV54800 | LBCR049-16 | LBP14372 | *Schizolecis guntheri* | Caraguatatuba-SP |
| **OTU 3** | LBPV54706 | LBCR021-16 | LBP14342 | *Schizolecis guntheri* | São Sebastião-SP |
| **OTU 3** | LBPV54707 | LBCR018-16 | LBP14342 | *Schizolecis guntheri* | São Sebastião-SP |
| **OTU 3** | LBPV54708 | LBCR020-16 | LBP14342 | *Schizolecis guntheri* | São Sebastião-SP |
| **OTU 3** | LBPV54709 | LBCR015-16 | LBP14342 | *Schizolecis guntheri* | São Sebastião-SP |
| **OTU 3** | LBPV54710 | LBCR013-16 | LBP14342 | *Schizolecis guntheri* | São Sebastião-SP |
| **OTU 3** | LBPV53375 | LBCR014-16 | LBP14335 | *Schizolecis guntheri* | São Sebastião-SP |
| **OTU 3** | LBPV53376 | LBCR016-16 | LBP14335 | *Schizolecis guntheri* | São Sebastião-SP |
| **OTU 3** | LBPV53377 | LBCR017-16 | LBP14335 | *Schizolecis guntheri* | São Sebastião-SP |
| **OTU 3** | LBPV53378 | LBCR080-16 | LBP14335 | *Schizolecis guntheri* | São Sebastião-SP |
| **OTU 3** | LBPV53379 | LBCR019-16 | LBP14335 | *Schizolecis guntheri* | São Sebastião-SP |
| **OTU 3** | LBPV61126 | LBCR078-16 | LBP14433 | *Schizolecis guntheri* | São Sebastião-SP |
| **OTU 3** | LBPV61127 | LBCR081-16 | LBP14433 | *Schizolecis guntheri* | São Sebastião-SP |
| **OTU 3** | LBPV61128 | LBCR079-16 | LBP14433 | *Schizolecis guntheri* | São Sebastião-SP |
| **OTU 3** | LBPV61129 | LBCR077-16 | LBP14433 | *Schizolecis guntheri* | São Sebastião-SP |
| **OTU 3** | LBPV61130 | LBCR076-16 | LBP14433 | *Schizolecis guntheri* | São Sebastião-SP |
| **OTU 3** | LBPV53279 | LBCR024-16 | LBP14310 | *Schizolecis guntheri* | Bertioga-SP |
| **OTU 3** | LBPV53315 | LBCR025-16 | LBP14319 | *Schizolecis guntheri* | Bertioga-SP |
| **OTU 3** | LBPV53317 | LBCR022-16 | LBP14319 | *Schizolecis guntheri* | Bertioga-SP |
| **OTU 3** | LBPV53318 | LBCR023-16 | LBP14319 | *Schizolecis guntheri* | Bertioga-SP |
| **OTU 4** | LBPV79547 | LBCR065-16 | LBP20209 | *Schizolecis guntheri* | Cajati-SP |
| **OTU 4** | LBPV79548 | LBCR066-16 | LBP20209 | *Schizolecis guntheri* | Cajati-SP |
| **OTU 4** | LBPV79549 | LBCR067-16 | LBP20209 | *Schizolecis guntheri* | Cajati-SP |
| **OTU 4** | LBPV79550 | LBCR068-16 | LBP20209 | *Schizolecis guntheri* | Cajati-SP |
| **OTU 4** | LBPV79551 | LBCR069-16 | LBP20209 | *Schizolecis guntheri* | Cajati-SP |
| **OTU 5** | LBPV61111 | LBCR082-16 | LBP14427 | *Schizolecis guntheri* | Angra dos Reis-RJ |
| **OTU 5** | LBPV61113 | LBCR027-16 | LBP14427 | *Schizolecis guntheri* | Angra dos Reis-RJ |
| **OTU 5** | LBPV61114 | LBCR026-16 | LBP14427 | *Schizolecis guntheri* | Angra dos Reis-RJ |
| **OTU 5** | LBPV61131 | LBCR083-16 | LBP14700 | *Schizolecis guntheri* | Angra dos Reis-RJ |
| **OTU 5** | LBPV61132 | LBCR029-16 | LBP14700 | *Schizolecis guntheri* | Angra dos Reis-RJ |
| **OTU 5** | LBPV61133 | LBCR031-16 | LBP14700 | *Schizolecis guntheri* | Angra dos Reis-RJ |
| **OTU 5** | LBPV61134 | LBCR030-16 | LBP14700 | *Schizolecis guntheri* | Angra dos Reis-RJ |
| **OTU 5** | LBPV61135 | LBCR028-16 | LBP14700 | *Schizolecis guntheri* | Angra dos Reis-RJ |
| **OTU 5** | LBPV60596 | LBCR035-16 | LBP14421 | *Schizolecis guntheri* | Parati-RJ |
| **OTU 5** | LBPV60597 | LBCR034-16 | LBP14421 | *Schizolecis guntheri* | Parati-RJ |
| **OTU 5** | LBPV60598 | LBCR033-16 | LBP14421 | *Schizolecis guntheri* | Parati-RJ |
| **OTU 5** | LBPV60599 | LBCR032-16 | LBP14421 | *Schizolecis guntheri* | Parati-RJ |
