# Supplementary material for: Genetic and Morphological Analyses Demonstrate That Schizolecis guntheri (Siluriformes: Loricariidae) Is Likely to Be a Species Complex
Source: Front Genet. 2018 Mar 2;9:69. doi: 10.3389/fgene.2018.00069 (PMC5841391; doi:10.3389/fgene.2018.00069)
Supplement: Supplementary file 3 [file Table_3.DOCX]

**Supplementary table 3.** Voucher numbers and total number of samples used in Principal Component Analysis (PCA).

| Locality | Drainages | Voulcher | Number of samples |
| --- | --- | --- | --- |
| Silva Jardim-RJ | São João basin | LBP18473 | 5 |
| Bom Jardim-RJ | Paraiba do Sul basin | LBP10759 | 10 |
| Angra dos Reis-RJ | Rivers of Angra dos Reis | LBP14427  LBP14700 | 5  5 |
| Ubatuba-SP | Rivers of Ubatuba | LBP3546  LBP7921 | 5  5 |
| Caraguatatuba-SP | Rivers of Caraguatatuba | LBP14372  LBP14384 | 2  5 |
| São Sebastião-SP | Rivers of São Sebastião | LBP14342  LBP14433 | 5  5 |
| Bertioga-SP | River of Bertioga | LBP14319 | 10 |
| Cajati-SP | Ribeira de Iguape basin | LBP20209  LBP898 | 5  5 |
| Morretes-PR | Rivers of Paranaguá bay | LBP3241  LBP3239  LBP7169  LBP2514 | 6  1  1  2 |
